# *Borrelia miyamotoi*—An Emerging Human Tick-Borne Pathogen in Europe

**DOI:** 10.3390/microorganisms9010154

**Published:** 2021-01-12

**Authors:** Katarzyna Kubiak, Magdalena Szczotko, Małgorzata Dmitryjuk

**Affiliations:** 1 Department of Medical Biology, Collegium Medicum, School of Public Health, University of Warmia and Mazury in Olsztyn, Zolnierska 14c, 10-561 Olsztyn, Poland; katarzyna.kubiak@uwm.edu.pl; 2 Department of Biochemistry, Faculty of Biology and Biotechnology, University of Warmia and Mazury in Olsztyn, Oczapowskiego 1A, 10-719 Olsztyn, Poland; magdalena.szczotko@uwm.edu.pl

**Keywords:** *Borrelia miyamotoi*, tick vectors, reservoir, *Borrelia miyamotoi* disease (BMD), BMD symptoms

## Abstract

*Borrelia miyamotoi* is classified as a relapsing fever spirochete. Although *B. miyamotoi* is genetically and ecologically distinct from *Borrelia burgdorferi* sensu lato, both microorganisms are transmitted by the same *Ixodes* tick species. *B. miyamotoi* was detected in *I. persulcatus* ticks in 1994 in Japan. A phylogenetic analysis based on selected sequences of *B. miyamotoi* genome revealed genetic differences between isolates from Asia, North America, and Europe, which are clearly separated into three genotypes. Symptomatic human cases of *Borrelia miyamotoi* disease (BMD) were first reported in 2011 in Russia and then in North America, Europe, and Asia. The most common clinical manifestation of BMD is fever with flu-like symptoms. Several differences in rare symptoms (thrombocytopenia, monocytosis, cerebrospinal fluid pleocytosis, or symptoms related to the central nervous system) have been noted among cases caused by Asian, European, and American types of *B. miyamotoi*. BMD should be considered in the diagnosis of patients after tick bites, particularly with meningoencephalitis, without anti-*Borrelia* antibodies in the cerebrospinal fluid. This review describes the biology, ecology, and potential of *B. miyamotoi* as a tick-borne pathogen of public health concern, with particular emphasis on Europe.

## 1. Introduction

In Europe, tick-borne diseases transmitted by *Ixodes ricinus* are the most common zoonoses with significant medical and veterinary importance [1]. This hematophagous arthropod is a reservoir and vector of many pathogenic microorganisms, including the bacteria *Borrelia burgdorferi* sensu lato (s.l.) complex—the causative agent of Lyme borreliosis (LB), *Rickettsia* spp., and *Anaplasma* spp., as well as the flavivirus responsible for tick-borne encephalitis (TBE) and the etiological protozoan agents of babesiosis [2,3]. With advanced methods of molecular biology, new tick-borne microorganism species and their genetic variants with confirmed or potential pathogenicity for humans and animals are still being identified [4]. One of the emerging *Ixodes*-borne diseases in the northern temperate climate zones of the world, including Europe, is *Borrelia miyamotoi* disease (BMD), caused by spirochete from the relapsing fever (RF) group of *Borrelia* [5,6]. Since 1994, when *B. miyamotoi* was first isolated from questing *I. presulcatus* ticks and mouse *Apodemus argentus* in Japan [7], it was considered to be a non-pathogenic endosymbiont. However, since 2011 many symptomatic *B. miyamotoi* infections in humans have been noted in Asia, North America, and Europe [8,9,10,11,12,13,14].

This review presents data on the biology, ecology, and the potential of *B. miyamotoi* as a human tick-borne pathogen of public health concern, with particular emphasis on Europe.

## 2. Review 

### 2.1. Taxonomic Position

*B. miyamotoi* is a Gram-negative bacteria included in the genus *Borrelia* from the family *Spirochaetaceae*, within the phylum *Spirochaetes* and the order *Spirochaetales* [15]. *Borrelia* species are obligate parasites, transmitted by arthropod vectors to vertebrate hosts. The biological feature that distinguishes *B. miyamotoi* and several other relapsing fever species from *B. burgdorferi* s.l. is transovarial transmission [16].

The *Borrelia* spirochete cells are 0.2–0.5 mm in diameter by 3–30 mm in length, with 15–20 periplasmic flagella (endoflagella) located in the periplasmic space between the outer membrane and the protoplasmic cylinder. These cells can move actively with frequent reversal of direction [15,17]. Due to limited *B. miyamotoi* biosynthetic potential, its in vitro culture is difficult (as other *Borrelia* species) and requires microaerophilic conditions and complex nutrition. However, it can be propagated in Kelly-Pettenkofer medium with fetal calf serum (MKP-F) [18]. 

Although the *Borrelia* species share spirochetal morphology, they have different biological, clinical, and epidemiological features. Based on their arthropod vectors and genetic characteristics two major groups of *Borrelia* were distinguished. The first group contains 20 *Borrelia* species, including the *B. burgdorferi* s.l. complex, an agent of LB, and are transmitted by *Ixodes* hard ticks. The second group includes 25 *Borrelia* species associated with human RF and mostly found in soft ticks (*Argasidae*) but also in lice (*B. recurensis*) and hard ticks (*B. miyamotoi*, *B. lonestari*, *B. theileri*). In RF-*Borrelia* complex only *B. miyamotoi* is transmitted by *Ixodes* ticks—a vector of *B. burgdorferi* s.l. complex [15,19,20]. These two groups are genetically similar but form distinct, independent monophyletic clades and share a common ancestor. In 2014, Adeolu and Gupta [21] proposed splitting the spirochetes from the genus *Borrelia* into two separate genera: a novel genus, *Borreliella* gen. nov., containing the causative agents of Lyme disease and a revised genus *Borrelia*, with spirochetes causing RF, including *B. *miyamotoi**. However, the proposed change in the name of this pathogenic bacteria species proved controversial and did not receive support among scientists, clinicians or public health authorities, who felt it would lead to confusion and pose a risk to patient safety [20,22,23].

### 2.2. Genome Organization and Genetic Diversity

The first information about the organization of the *B. miyamotoi* genome and its differences in relation to the known species from the LB- and RF-*Borrelia* groups was published in 1995 [7]. Later, more advanced molecular analysis of Asian, American, and European *B. miyamotoi* isolates from *Ixodes* ticks and clinical samples revealed the complexity of the genome structure typical of *Borrelia* spirochetes [24,25,26,27,28,29]. However, the most information was obtained by sequencing the genome of *B. miyamotoi* Izh-4 isolate from a Russian patient [30]. The complete genome of a single *B. miyamotoi* cell consists of one linear chromosome (~900 kb) and 12 linear and two circular plasmids (from 6 to 73 kb). Two of the plasmids (lp70 and lp64) had not previously been found in other *Borrelia* species. A total of 1362 genes, including 1222 protein-coding genes, 103 pseudogenes, 31 genes for transfer RNA (tRNA), a cluster of three genes of ribosomal RNA (rRNA), and three genes of non-coding RNA (ncRNA) were identified. In *B. miyamotoi* virulence, a significant role is played by plasmid lp4, which includes genes of variable membrane proteins (VMPs), necessary to mask the bacteria from the host immune system and prolong the infection [30,31,32]. A comparison of different *B. miyamotoi* isolates revealed that the number and order of VMPs genes were unique for each of them [30]. 

Phylogenetic analysis based on genome sequences of *B. miyamotoi* showed genetic differences between isolates from Asia, North America and Europe which are clearly separated into three types (genotypes) and form a monophyletic clade inside the RF-*Borrelia* spirochetes [30]. However, the genetic differences between the *B. miyamotoi* isolates are probably not connected with geographic origin, but rather with pathogenicity, vector competence, and host range [16,24]. 

**Figure 1:**
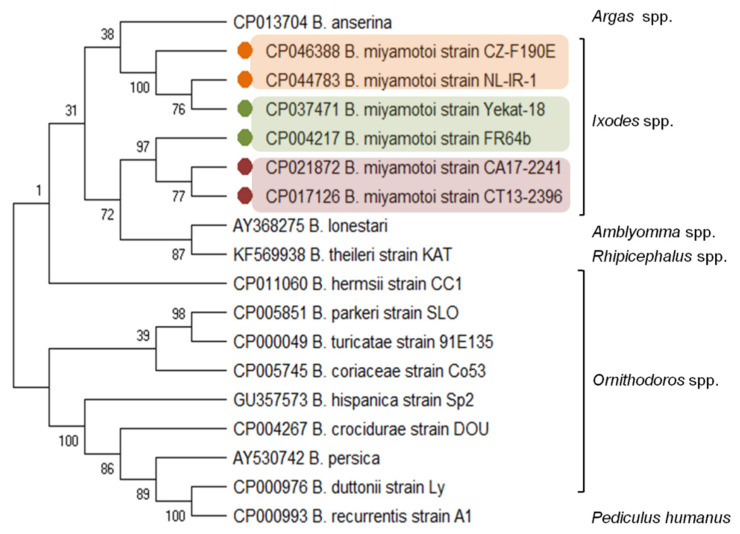
Molecular relationships between *B. miyamotoi* and other RF *Borrelia* species based on the sequences of the *glpQ* gene selected from GenBank. The consensus tree constructed using the neighbor-joining method and the maximum composite likelihood as the distance method; numbers at the tree nodes indicate bootstrap value from 1000 replicates; analyses were conducted in MEGA X [38]. Marks: orange—European type, green—Asian type, red—American type *of B. miyamotoi*. The genus names of the vectors were added.

The *B. miyamotoi* genetic distance from other LB species and the relationship with the species from the RF group is evidenced by the carriage and expression of a *glpQ* gene, coding the immunoreactive protein glycerophosphodiester phosphodiesterase [33,34]. The *glpQ* gene and GlpQ protein are conserved among the members of the genus *Borrelia*, except LB spirochetes (Figure 1). Therefore, GlpQ is usually used as a marker in molecular and serological tests to detect RF spirochete infections and to distinguish cases of LB and other tick-borne infections (e.g., anaplasmosis, babesiosis) [8,35,36,37].

### 2.3. Vectors and Reservoirs

Since the first detection in 1994 in questing adult *I. persulcatus* in Japan [7], *B. miyamotoi* has been recorded in *Ixodes* ticks from many countries in Asia (Russia, Japan, China, Mongolia, Korea), North America (USA, Canada), and Europe. In Asia, the main vector of *B. miyamotoi* is *I. persulcatus*, which is also detected in *I. ovatus, I. pavlovskyi*, *I. nipponensis,* and *Haemaphysalis concinna* [7,39,40,41,42]. *I. pacificus* is known as a *B. miyamotoi* vector in the western USA and *I. scapularis* in the north-central USA and Canada [43,44,45,46,47]. In Europe, vector competence for *B. miyamotoi* has been demonstrated for *I. ricinus* and *I. persulcatus* [12,36,45,48,49,50,51,52,53,54,55,56,57,58,59,60,61,62,63,64,65,66,67,68,69,70,71,72,73,74,75,76,77,78,79,80,81] (Table 1). Worldwide, *B. miyamotoi* prevalence in questing *Ixodes* ticks ranges from 0.2 to 10% [42,43,44,77,82]. This pathogen has been detected in all three tick life stages (larvae, nymphs and adults) [68,83,84]. In European populations of *I. ricinus*, *B. miyamotoi* was identified in 0.1–2% larvae [78,85], 0.4–2.8% nymphs [12,36,78,79] and 3.0–4.3% of adults [78,79]. 

The relatively high percentage of naturally infected *I. ricinus* larvae is an effect of well-documented *B. miyamotoi* efficient transovarial (vertical) transmission from female ticks to their offspring [43,83]. Van Duijvendijk et al. [85] also reported that the larvae of *I. ricinus* can transmit *B. miyamotoi* into nymphs. Transmission and acquisition of the pathogen from rodent hosts to larvae is possible [54]. *Ixodes* nymphs and adults can also be naturally infected by *B. miyamotoi* during feeding on vertebrate hosts [85]. 

Competence as a *B. miyamotoi* reservoir was demonstrated for *Apodemus* spp. mice, *Myodes glareolus* (the bank vole), and *Peromyscus leucopus* (the *white-footed mouse*) [43,86]. However, DNA detection of spirochetes in mammals (e.g., squirrels, raccoons, hedgehogs, wild boar, roe deer) and birds (e.g., blackbirds, European robins, European greenfinches, wild turkeys) did not exclude these species as competent reservoirs [6,82,87,88]. 

Despite demonstrated effective routes of *B. miyamotoi* transmission, the low infection rate of *Ixodes* ticks is still unclear. This can be explained by the negative effect of spirochetes on the survival rate of infected ticks or by the low rate of ticks acquired during feeding on infected hosts [44,89]. Moreover, laboratory and field studies revealed that *B. miyamotoi* does not cause a persistent infection in wild rodents and probably provokes the production of antibodies against *B. miyamotoi,* making the rodents resistant to infections [85,90]. This may indicate that wild rodents are able to eliminate *B. miyamotoi* and do not play a significant role in its spread. However, this does not change the fact that *B. miyamotoi* is constantly present in *Ixodes* tick populations and can infect humans in all life stages.

### 2.4. Borrelia miyamotoi Disease (BMD) in Europe

*B. miyamotoi* is increasingly documented as a human pathogen, especially in the northern hemisphere of the world, where it is co-circulated with *B. burgdorferi* s.l., bacterium causing LB. Both use the same hard tick species as vectors [6]. In 2011, the first series of 46 patients with febrile diseases caused by *B. miyamotoi* was described in Yekaterinburg, in the Asian part of Russia [8]. Evidence of *B. miyamotoi* human infection was then confirmed in the USA, Japan, and China [5,9,10,11,40,91,92]. 

In Europe, several single cases of BMD have been described [13,14,93,94,95,96]. Human cases of a positive PCR for *B. miyamotoi* in Europe are summarized in Table 2. BMD is usually manifested by several episodes of fever (~40 °C) and flu-like symptoms [8]. However, symptoms of *B. miyamotoi* disease are very often non-specific. Meningoencephalitis is one of the potentially dangerous consequences in the course of BMD. In 2013, Hovius et al. [13] reported, for the first time in Europe, meningitis caused by *B. miyamotoi* infection in an immunocompromised patient (Table 2). A similar case of an immunocompromised individual from New Jersey, USA, was described the same year [92]. Another two cases of meningitis during human BMD were diagnosed in Sweden in 2018. Particularly noteworthy is that, in one of these cases, central nervous system (CNS) symptoms were first diagnosed in an immunocompetent patient [94] (Table 2). Symptoms of BMD were also noted in a seropositive immunocompetent patient in the Netherlands. In this patient, *B. miyamotoi*–specific PCR of the blood was negative. Moreover, testing for anti-GlpQ and the anti–variable major proteins (VMPs) IgM and IgG using ELISA and Western blot in serum samples demonstrated a clear seroconversion, predominantly for IgG against GlpQ [37]. In turn, BMD-associated neuroborreliosis in an immunocompromised patient was found in 2015 in Germany [93]. In a preliminary report, one case of BMD was demonstrated in the course of neuroborreliosis in Poland [96]. A human case of *B. miyamotoi* infection was diagnosed in Austria as well (Table 2). Although a phylogenetic analysis of the *B. miyamotoi* isolate indicated an infection by a European type, the patient claimed a tick bite in the United States. Therefore, this case origin is unclear [95]. Additionally, one blood sample in the Netherlands was found to be PCR-positive and the patient reported no symptoms of BMD but had erythema migrans [14] (Table 2). Due to the coexistence of *B. burgdorferi* s.l. and *B. miyamotoi* spirochetes, the correct diagnosis of LB and BMD may cause many difficulties caused by the overlapping manifestation of symptoms of both diseases.

An increase in *B. miyamotoi* infections has been recently recorded during screening tests. A positive Real-Time PCR for *B. miyamotoi* in the blood from 43 symptomatic patients was revealed in 2020 (Table 2). These studies included 824 patients in expressing signs and symptoms compatible with a persistent polymorphic syndrome, possibly due to a tick bite (neurological/musculoskeletal pain, and cognitive dysfunction, sleep disturbance, and fatigue, lasting for at least six months) and living in different regions of France. According to the authors of the study, the data suggest that *B. miyamotoi* infection may be persistent and long-term [97]. 

Few studies in Europe have dealt with the serological evidence of *B. miyamotoi*. This exposure was found among forestry workers in the Netherlands and febrile patients in Alsace, France [65,98]. More recently, a case of post-tick bite febrile syndrome has been reported in western Europe and serological results suggest that *B. miyamotoi* was the causative agent of the patient’s symptoms [37]. Reiter et al. [99] studied difference in seroprevalence between distinct populations in Austria by various immunoblotting methods. Antibodies were detected in the sera of 7/53 hunters and in 1/11 sera of Lyme neuroborreliosis patients, 17/74 sera of cases with high concentrations of anti-*B. burgdorferi* s.l. (α-Bbsl), 7/50 in α-Bbsl negative cases, 5/14 in healthy blood donors from commercial suppliers, and 10/35 from the Austrian Red Cross workers. In the same studies, GlpQ serology was negative in two PCR-positive cases.

### 2.5. Clinical Manifestation

A diagnosis of BMD should be considered in patients who experience fever attacks and live or spend time in regions where environmental conditions are favorable for ticks and their hosts. [95]. *B. miyamotoi* infection does not present specific symptoms of the relapsing fever group. Typically, patients experience fever with flu-like symptoms such as chills, headaches, muscle, and joint aches and general fatigue [89,95]. In the available literature, cases with recurrent febrile episodes interspersed with fever-free periods characterizing classic relapsing fever have been rarely reported [6,8,16]. In cases reported in Russia, up to three fever episodes were observed [8]. However, since patients usually receive antibacterial treatment after diagnosis the number of registered febrile episodes may be underestimated. In the case of BMD, there are no other typical symptoms of recurrent fever caused by other spirochetes of this group, such as epistaxis, abortion, jaundice, or severe organ failure. The common features of *B. miyamotoi* infection and classic relapsing fever include headache, chills, muscle aches, joint pain, and nausea/vomiting [6]. 

**Figure 2:**
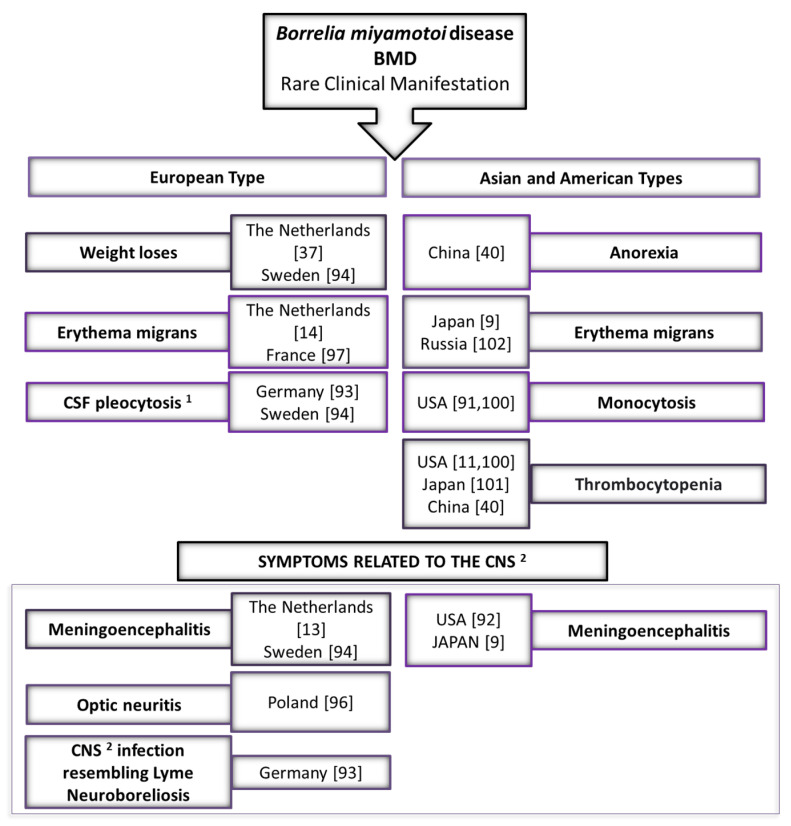
Rare clinical manifestations of *Borrelia miyamotoi* disease (BMD)—comparing the European type with Asian (Siberian) and American types. Notes: ^1^ CSF—cerebrospinal fluid; ^2^ CNS—central nervous system.

Several differences in rare clinical presentation have been noted between cases caused by Asian, European and American types of *B. miyamotoi* (Figure 2). One of them is cytopenia (especially thrombocytopenia) which has not been observed in patients infected by the Asian type of *B. miyamotoi* in Russia, but was recorded in half of the American cases [11,100]. On the other hand, thrombocytopenia was reported in a patient on the island of Hokkaido in Japan, where the Asian type of *B. miyamotoi* should be genetically close to the Russian isolate. However, BMD was confirmed only by serological test in this case [101]. Increased leukocyte count and thrombocytopenia also occurred occasionally in patients diagnosed with BMD in Northeastern China [40]. A characteristic symptom of the infection of the American type is also monocytosis [91,100]. Erythema migrans, also present in LB, was recorded in Russian and Japanese patients, in which the infection was caused by the Asian type of *B. miyamotoi* [9,102], as well as in the case of the European type of *B. miyamotoi* in the Netherlands and France [14,97] (Figure 2). 

Meningoencephalitis is a common complication in the course of BMD of all three *B. miyamotoi* types. Cases of meningitis have been reported in the USA, Japan, and in Europe (the Netherlands and Sweden) [9,13,92,94]. Some of the symptoms characteristic for BMD caused by the European type have been observed (Figure 2). For example, two patients lost weight. In the case of a 72-year-old female patient from the Netherlands, it was 2.5 kg within three weeks of a tick bite, while a 66-year-old woman from Stockholm (Sweden) lost 15 kg within six months of illness [37,94] (Figure 2). Although Chinese patients diagnosed with Siberian *B. miyamotoi* infections have reported anorexia, there is no specific weight loss data available [40]. Cerebrospinal fluid (CSF) pleocytosis was observed in a patient in Germany and a patient in Sweden. Both analyses of CSF showed a total leucocyte count significantly above normal [93,94]. A preliminary report case of BMD has been recently reported in Poland. DNA of *B. miyamotoi* was detected in the CSF of a patient who had suffered for three months from blurred vision in the left eye. Although the patient did not report typical symptoms of BMD (such as relapsing fever) and did not indicate a tick bite habit in the medical interview, further diagnostics showed that the left eye exhibited extraocular optic neuritis. Brain magnetic resonance imaging (MRI) revealed hyperintense signal abnormalities in the white matter of the brain hemispheres. The optic nerve was thinned and obliterated, which was indicative of fibrosis of the nerve and its sheath. In addition, some demyelinating changes were found in both hemispheres. The authors of the report point to patients with neurological symptoms and questionable serological findings presenting a serious diagnostic problem. This indicates the need for further studies of patients with signs of central nervous system infection [96]. In Germany, another case of *B. miyamotoi* infection of the CNS resembling neuroborreliosis was investigated [93] (Figure 2).

It appears that co-infection of *B. miyamotoi* with other tick-borne pathogens does not exacerbate the symptoms of the disease. Patients diagnosed in China who were co-infected with *Candidatus* Rickettsia tarasevichiae and *Anaplasma capra* had no more complicated symptoms or prolonged course of BMD [40]. 

### 2.6. Diagnosis

The diagnosis of *B. miyamotoi* infection should always be considered in all patients who live or visit endemic areas in North America, Asia, and Europe [16]. Different mechanisms of infection transmission should be taken into account in distinguishing BMD from other tick-borne diseases. *B. miyamotoi* infection can be acquired in humans by a bite in any stage of tick development, including the larval stage due to transovarial transmission [41]. Common BMD symptoms, such as recurrent fever, flu-like symptoms, and fatigue are the most important in providing support for a diagnosis. The correct diagnosis can be misleading because patients with other tick-borne diseases, such as LB, human granulocytic anaplasmosis and babesiosis, have similar symptoms [16]. 

BMD diagnosis is possible through several methods, e.g., by blood smear, different types of PCR, determination of IgG and IgM antibodies, in vitro culture, and/or isolation by animal inoculation. However, two diagnostic methods have been most commonly used to detect *B. miyamotoi* infections in humans—*B. miyamotoi* DNA/RNA detection and serodiagnosis [82]. In Europe, enzyme-linked immunosorbent assay (ELISA) and Western-blot serodiagnostic tests and PCR tests (Table 2) are used, either in combination or separately, to detect *B. miyamotoi* spirochete infection in humans. In ELISA and a confirmatory Western blot, specific antibodies against GlpQ protein, as a non-reactive antigen from *B. burgdorferi* s.l. [65,94,98], in human serum [10,65,96,98] and in CSF samples [94] were used. Sometimes, however, GlpQ-based *B. miyamotoi* serology as a single marker does not hold specificity or sensitivity [13,99]. Therefore, searching for other antigens, such as the variable major proteins (VMPs) of *B. miyamotoi*, should be evaluated as diagnostic additional markers to ensure sufficient specificity for an accurate diagnosis [99]. In such cases, it appears that PCR methods are also more reliable. The amplification of various genes (*16S* rDNA, *fla*, *p66*, *16S–23S* internal spacer region) was used by the real-time PCR method in serum and CSF assays [13,95,103]. However, the *glpQ* gene was most often tested in combination with other genes or individually by qPCR. To determine the *B. miyamotoi* type, nested PCR [96] or sequencing [95,97] is used. 

### 2.7. Treatment and Prevention

*B. miyamotoi* infection is generally effectively treated with antibiotics following guidelines used for the treatment of LB [6]. In cases reported in Europe, doses of 200 mg doxycycline have been used successfully once or twice daily for two weeks [94,95]. Intravenous ceftriaxone 2000 mg once a day provided for two weeks has been effectively used in the case of meningoencephalitis diagnosed with BMD [13]. 

Unfortunately, since no vaccine against *Borrelia* spirochetes, including *B. miyamotoi*, has yet been developed or approved [104], it seems that the only effective preventive measures will be the same as for other diseases transmitted by *Ixodes* ticks, such as LB. These include personal protective measures to avoid tick bites, as well as environmental modification to reduce the number of ticks [16].

## 3. Conclusions

In Europe, BMD represents an emerging tick-borne disease with an increasing number of diagnosed cases in humans. In the last seven years, *B. miyamotoi* infection has been noted in both immunocompetent and immunocompromised patients. Fever and other flu-like symptoms suggest a mild infection course. However, serious symptoms related to the central nervous system can be observed. BMD should be considered in the diagnoses of patients after tick bites, particularly with meningoencephalitis, without anti-*Borrelia* antibodies in CSF. Currently, since there is no specific, reliable serological marker, serodiagnostics should be combined with molecular methods (such as different types of PCR) for a correct diagnosis.

## Figures and Tables

**Table 1:** *Borrelia miyamotoi* in host-seeking and feeding *Ixodes ricinus* and *Ixodes persulcatus* ticks in Europe.

Part of Europe	Country	*B. miyamotoi* Prevalence (%)	Reference
North	Denmark	0.2–1.3	[80]
	Estonia	0.4 (*I.r*.) ^1^; 2.7 (*I.p*.) ^2^	[68]
	Finland	0.56	[48]
	Latvia	1.1 (*I.r*.) ^1^; 1.27 (*I.p*.) ^2^	[76]
	Norway	0.9–1.3	[49,50]
	Sweden	0.7 (*B.m*.-like) ^3^	[51]
Central	Czech Republic	3.2	[45]
	Hungary	4.8	[52]
	Poland	0.5–3.9	[53,54,55,56]
	Romania	1.5	[57]
	Slovakia	0.75–1.0	[58,59]
	Switzerland	2.5	[60]
West	Ireland	10	[77]
	England	0.4 (N) ^4^–0.73	[12,81]
	France	1.2–2.2	[63,64,65]
	Belgium	1.1–2.4	[61,62]
	Germany	0.8–8.9	[66,67,78,79]
	The Netherlands	2.5 (N) ^4^–3.8	[36,61]
South	Italy	0.74 (N) ^4^	[69]
	Portugal	0.16	[70]
	Serbia	1.4 (N) ^4^	[71]
	Spain	0.6–1.0	[72,73,74]
	Turkey (European part)	0.4	[75]

^1^ (*I.r*.)—*Ixodes ricinus*; ^2^ (*I.p*.)—*Ixodes persulcatus*; ^3^ (*B.m*.-like)—*B. miyamotoi-like*; ^4^ (N)—nymphs.

**Table 2:** *Borrelia miyamotoi* disease (BMD) DNA-positive cases in Europe.

Country	Patient	Number of Cases, Percentage of Cases among Persons Studied	Case	Assay	Reference
The Netherlands	70-year-old man	1 single case	Meningoencephalitis	qPCR	[13]
The Netherlands	42-year-old man	1/626, 0.16%	EM ^1^, asymptomatic	qPCR	[14]
Germany	74-year-old woman	1 single case	Neuroborreliosis, Immunocompromised	qPCR	[93]
Sweden	53-year-old woman 66-year-old woman	2 single cases	Meningitis, Immunocompetent Meningitis, Immunocompromised	PCR	[94]
Poland	47-year-old man	1/133, 0.75%	Neuroborreliosis, patient with alcohol abuse	Nested PCR	[96]
Austria	51-year-old woman	1 single case	Symptomatic	qPCR	[95]
France	NA ^2^	43/824, 5.22%	Symptomatic	qPCR	[97]

^1^ EM, erythema migrans; ^2^ NA, not applicable.

## Data Availability

The data presented in this study are openly available.
